# Integrating Multiple‐Covariate Distance Sampling and Habitat Modeling to Inform Conservation of the Asian Houbara in Central Iran

**DOI:** 10.1002/ece3.72909

**Published:** 2026-01-30

**Authors:** Reyhaneh Miranzadeh‐Mahabadi, Mahmoud‐Reza Hemami, Hossein Bashari, Mostafa Abyareh, Mohsen Ahmadi

**Affiliations:** ^1^ Department of Natural Resources Isfahan University of Technology Isfahan Iran; ^2^ Central Laboratory Isfahan University of Technology Isfahan Iran

**Keywords:** anthropogenic threats, *Chlamydotis macqueenii*, habitat selection, line‐transect survey, population monitoring

## Abstract

Reliable estimates of abundance and habitat associations are critical for conserving low‐density species such as the Asian houbara ( *Chlamydotis macqueenii* ). Despite its vulnerable global status, robust estimates of houbara population size and habitat requirements remain scarce across much of its range. We combined multiple‐covariate distance sampling (MCDS) with habitat modeling (Random Forest, GAMs, and GLMs) to estimate density and identify habitat relationships of houbaras in central Iran. In spring 2022, 223 line‐transect surveys (1449 km) covering a 10,000 km^2^ area yielded 205 individuals across 67 detections. The best‐supported MCDS model included fine gravel cover (positive) and vegetation height (negative) as detectability covariates, though their effects were weak. This model estimated a density of 0.53 individuals/km^2^ (95% CI: 0.37–0.75), corresponding to ~5293 individuals (95% CI: 3778–7473). Estimates were nearly identical to those from the best conventional distance sampling (CDS) model, indicating that detectability covariates did not materially improve model accuracy. However, habitat models consistently identified fine gravel cover and vegetation height as the most influential predictors, underscoring their ecological relevance for habitat use. Results indicate an ongoing population decline relative to previous regional estimates, highlighting the need for continued monitoring. Integrating population estimation with habitat modeling provides a practical framework for improving conservation assessments of the Asian houbara and other ground‐dwelling birds in open habitats. Conservation actions should prioritize the protection and management of suitable habitats, supported by standardized survey protocols that improve population assessments and inform management decisions.

## Graphical abstract

We combined multiple‐covariate distance sampling (MCDS) with habitat modeling to estimate the abundance and habitat associations of the vulnerable Asian houbara ( *Chlamydotis macqueenii* ) in central Iran. Line‐transect surveys in spring 2022 yielded an estimated density of 0.53 individuals/km^2^ (~5293 birds), with nearly identical results from conventional distance sampling, indicating that detectability covariates did not substantially improve model accuracy. However, fine gravel cover and vegetation height consistently emerged as key predictors in habitat models, underscoring their ecological importance and demonstrating the value of integrating distance sampling with habitat modeling for conservation of low‐density species in arid ecosystems.

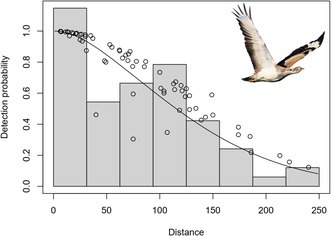

## 1 Introduction

The Asian houbara ( *Chlamydotis macqueenii* ), a cryptic and desert‐adapted bird, is undergoing marked population declines across its range. The geographic range extends from Egypt to Central Asia. Currently listed as Vulnerable by the IUCN and included in Appendix I of CITES, the species faces a complex array of conservation challenges (BirdLife International 2021). Despite its threatened status, estimates suggest that populations are fragmented and sparse, limiting the capacity to effectively evaluate conservation priorities. As of 2024, global estimates suggest a mature population between 33,000 and 67,000 individuals, with Kazakhstan—one of the primary breeding grounds—harboring nearly 49,000 birds (Riou et al. 2011). Anthropogenic pressures continue to pose serious risks, especially during the non‐breeding season (Kessler et al. 2024). While traditional falconry in the Middle East was once sustainable, the proliferation of advanced hunting technologies has greatly intensified the threat to wild populations. Illegal hunting, live capture, and smuggling are now widespread in critical wintering areas, particularly in Pakistan, Afghanistan, and Iran (Burnside et al. 2018). At the same time, breeding grounds in countries such as Kazakhstan and Uzbekistan continue to experience high mortality rates due to hunting and trapping (Combreau et al. 2001). Conservation efforts for the Asian houbara have followed two main approaches. Early ex situ captive‐breeding programs, initiated in the 1970s, enabled large‐scale releases but have shown limited long‐term effectiveness due to low survival and behavioral differences in captive‐reared birds (Mendelssohn et al. 1979; Azar et al. 2018; Dolman et al. 2021). More recent in situ strategies emphasize habitat protection, management, and policy enforcement to sustain wild populations (Combreau et al. 2025). In Iran, conservation measures include legal protection of the species and its habitats, with more than 60 protected areas supporting wintering or breeding populations. However, systematic population monitoring remains limited, and many houbara occur outside protected areas, particularly during migration. The absence of standardized, large‐scale population assessments has constrained evaluations of conservation effectiveness and the prioritization of management actions, underscoring the need for robust abundance estimates and habitat‐based analyses such as those presented in this study. Robust estimates of population size and density are foundational for conservation planning, as they inform both IUCN assessments and targeted management actions (Le Breton et al. 2019; Miller et al. 2007). While previous research has explored aspects of houbara density and habitat use in various parts of its range (Launay 1999; Carrascal et al. 2006, 2008; Hingrat et al. 2008; Chammem et al. 2012), large‐scale, systematic studies from Iran—particularly its central regions, which serve as vital wintering habitats—are notably lacking. Although localized studies exist (e.g., Aghanajafizadeh et al. 2012), the absence of broad‐scale monitoring hampers our ability to track trends and implement effective conservation interventions.

Estimating the abundance of ground‐dwelling birds like the houbara presents unique methodological challenges, often requiring tailored approaches. Several techniques have been used for both Asian and African houbaras ( *C. undulata* ), including point counts (Le Cuziat et al. 2005), capture‐mark‐recapture (Combreau et al. 2001), and distance sampling (Aghanajafizadeh et al. 2012; Koshkin et al. 2014; Monnier‐Corbel et al. 2022). Among these, line‐transect distance sampling is generally preferred in large, open landscapes due to its ability to account for detectability, particularly for elusive species like the houbara that rely on camouflage and typically flush at short distances (Kessler et al. 2024; Buckland et al. 2015).

Across its wide geographic range, the Asian houbara occupies heterogeneous habitats and faces region‐specific threats, resulting in strong spatial variation in population density and trends. Consequently, effective conservation planning relies on regionally robust estimates rather than extrapolation from limited sites. The present study focuses on a 10,000 km^2^ area in central Iran that represents an important wintering and migratory habitat within the species' broader range, providing region‐specific baseline data that contribute to national and range‐wide conservation assessments.

Here, we present the first large‐scale assessment of Asian houbara density in central Iran, covering an area of 10,000 km^2^. The objectives of this study are to: (1) estimate population density and abundance to assess the species' conservation status; (2) evaluate the influence of environmental and survey‐related covariates on detectability and density; and (3) identify key microhabitat features that influence habitat selection. Our findings provide critical baseline data to support conservation strategies at both national and regional scales.

## 2 Methods

### 2.1 Study Area

**FIGURE 1:**
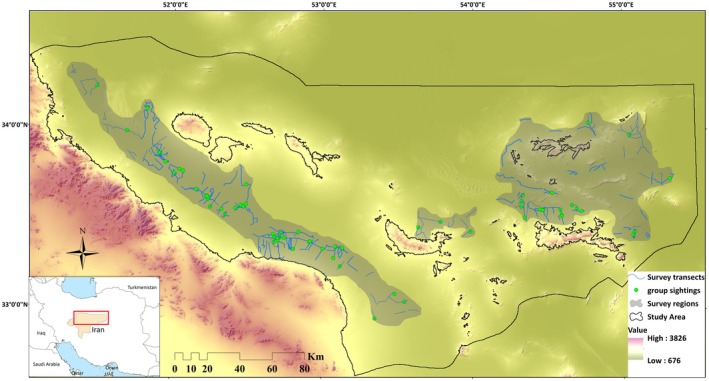
Location of the study area in Isfahan Province, central Iran, where distance sampling efforts were conducted between 2022 and 2023 to estimate the density of the Asian houbara. The map displays group sightings and sampling transects within the survey region.

This study was carried out in the arid plains of eastern Isfahan Province, situated on Iran's central plateau. The broader region covers approximately 47,758 km^2^ (29°55′ to 33°59′N, 55°29′ to 51°09′E), from which a 10,000 km^2^ subset was selected for detailed analysis based on ecological suitability, accessibility, and known species presence (Figure 1). Areas deemed unsuitable—such as steep slopes (> 15°), salt flats, and barren lands—were excluded from the sampling frame.

The region experiences a warm, arid climate, with an average annual temperature of 18.5°C and precipitation ranging from 82 to 192 mm (WorldClim 2; Fick and Hijmans 2017). Elevations span 685–1500 m (Copernicus Digital Elevation Model; European Space Agency 2019), creating a diverse landscape of mountains, hills, plains, deserts, and sand dunes interspersed with human‐modified environments including settlements, farmlands, mining areas, and transportation infrastructure. Vegetation is predominantly sparse, with xerophytic shrubs such as *Artemisia sieberi*, *Seidlitzia rosmarinus*, *Haloxylon aphyllum*, and *Zygophyllum eurypterum*. Livestock grazing is widespread, primarily involving sheep, goats, and camels. The area also supports native predators such as caracals ( *Caracal caracal* ), golden jackals ( *Canis aureus* ), red foxes ( *Vulpes vulpes* ), and golden eagles ( *Aquila chrysaetos* ) (Darvishsefat 2006; Pakniat et al. 2021). This ecologically diverse habitat serves as an important wintering and migratory zone for the target species.

### 2.2 Sampling Design and Data Collection

Field surveys were conducted over two consecutive winters (2022–2023) using line‐transect distance sampling. The study area was mainly open desert, though small patches of sparse *Haloxylon* woodland were present. These were not as open as the surrounding desert, but canopy cover was low and houbaras remained readily detectable within them. Based on prior habitat assessments and expert consultation with the Iranian Department of Environment, the study area was stratified into two zones: a high‐density stratum (3000 km^2^) and a low‐density stratum (7000 km^2^). Transects were approximately 7 km long, with randomly generated starting points created in ArcGIS to ensure spatial independence and comprehensive area coverage (Figure 1). Surveys were conducted between mid‐November and mid‐February, aligning with the peak wintering period. Each transect was surveyed by a two‐person team from a vehicle between 08:00 and 16:00 at a constant speed of 20–30 km/h. One observer recorded sightings with a GPS unit and a handheld laser rangefinder, while the driver maintained a steady speed. For each observation, data collected included transect coordinates (start/end), group size, observation angle (bearing), perpendicular distance from the transect line, and environmental variables such as wind, cloud cover, and visibility. Key assumptions of distance sampling include that groups are recorded at their initial location, prior to any movement in response to the observer, and that the probability of detection on the transect line (i.e., at zero distance) is equal to one (see Buckland et al. 2001; Thomas et al. 2002 for a complete list of assumptions). To meet these assumptions, observers marked the position of each group at first sighting and subsequently measured the required distances. To minimize the risk of double‐counting, each group was logged with GPS at the point of first detection, and when birds flushed, their flight direction was noted to ensure subsequent sightings along the same transect represented distinct groups. Perpendicular distances were calculated using basic trigonometry. To ensure accuracy and consistency in detection probability, surveys were suspended during adverse weather conditions such as heavy rainfall or strong winds.

### 2.3 Microhabitat Characterization

To evaluate fine‐scale habitat associations, microhabitat data were collected immediately after each bird detection, ensuring that the measurements reflected the conditions actually experienced by the species. At each detection point, one 5 × 5 m quadrat was established (Carrascal et al. 2008). Within quadrats, vegetation cover, shrub/bush percentage, and mean vegetation height (measured with a 3‐m graduated rod; Ucero et al. 2025) were recorded. Soil composition was characterized using three subsamples, categorized into coarse gravel (19–38 mm), medium gravel (8–19 mm), and fine gravel (3–8 mm), measured directly with a tape. These size classes reflect ecologically meaningful differences influencing water infiltration, vegetation establishment, and concealment opportunities.

### 2.4 Distance Sampling Analysis

Population density was estimated using the Distance package in R (Miller and Thomas 2015; R Development Core Team 2010) under the CDS framework (Buckland et al. 2001; Thomas et al. 2010). The detection function—which models the probability of observing a group based on its distance from the transect—was fitted using three candidate key functions: half‐normal, uniform, and hazard‐rate. Observations were truncated at the 95th percentile of detection distances, and sightings beyond 250 m were excluded, as beyond this distance observations were sparse and detection probability dropped below ~0.15, creating a long tail that reduced model stability (Buckland et al. 2001). This cutoff ensured the detection function was well defined in the core range while aligning with the local scale of houbara habitat use and field visibility conditions.

Model selection was based on Akaike's Information Criterion (AIC) (Buckland et al. 1997), while goodness‐of‐fit was assessed through Quantile–Quantile (Q–Q) plots and the Cramér–von Mises (C–vM) test. To account for variation in detection probability, MCDS models were developed (Miller et al. 2019), incorporating covariates such as vegetation cover, vegetation height, group size, and soil composition. To minimize redundancy, only covariates with low pairwise correlation (Pearson's *r* < 0.7) were retained for model inclusion. Model selection was guided by minimization of AIC in combination with inspection of goodness‐of‐fit diagnostics and biological plausibility. When candidate models were within 2 AIC units, we further considered detection‐function plots, parameter estimates, and ecological interpretability before selecting the best‐supported model. Model averaging was not undertaken, as the best‐supported MCDS model (AIC = 703.43) was > 2 units lower than the closest CDS alternative (AIC = 705.01), indicating stronger support for MCDS under the AIC criterion.

### 2.5 Predictive Modeling of Presence and Density

To identify key environmental drivers of houbara density, we used three complementary modeling approaches, each addressing different aspects of data structure. Generalized Linear Models (GLMs) were applied to assess linear relationships between predictors and density, assuming a Poisson distribution. While GLMs provide interpretable coefficients, they are limited in handling nonlinear patterns. Generalized additive models (GAMs) extended this framework by incorporating smoothing functions to capture nonlinear responses, offering greater flexibility but requiring careful control of model complexity (Guisan and Zimmermann 2000). Random forests (RFs), a non‐parametric ensemble method, were used to model complex interactions and non‐linearities without assuming a specific data distribution. RFs are robust to multicollinearity and include internal validation procedures to reduce overfitting (Liaw and Wiener 2002), although they lack the parametric interpretability of GLMs and GAMs. Together, these models offered a balanced approach, combining interpretability, flexibility, and predictive accuracy to robustly assess environmental influences on houbara distribution. All models were implemented using the caret package (Kuhn 2008) in R. Model performance was evaluated using the mean *R* ^2^ from 10‐fold cross‐validation. Hyperparameters were optimized via grid search to improve predictive accuracy. Prior to analysis, pairwise correlations among predictor variables were assessed using Pearson's correlation coefficient. When two variables were strongly correlated (*r* > 0.7), the one considered less ecologically informative was excluded to reduce redundancy and potential collinearity in the models. Model interpretation was supported by response curves and partial dependence plots, which provided insights into how each predictor influenced species presence and density (Friedman 2001).

## 3 Results

### 3.1 Population Surveys

**FIGURE 2:**
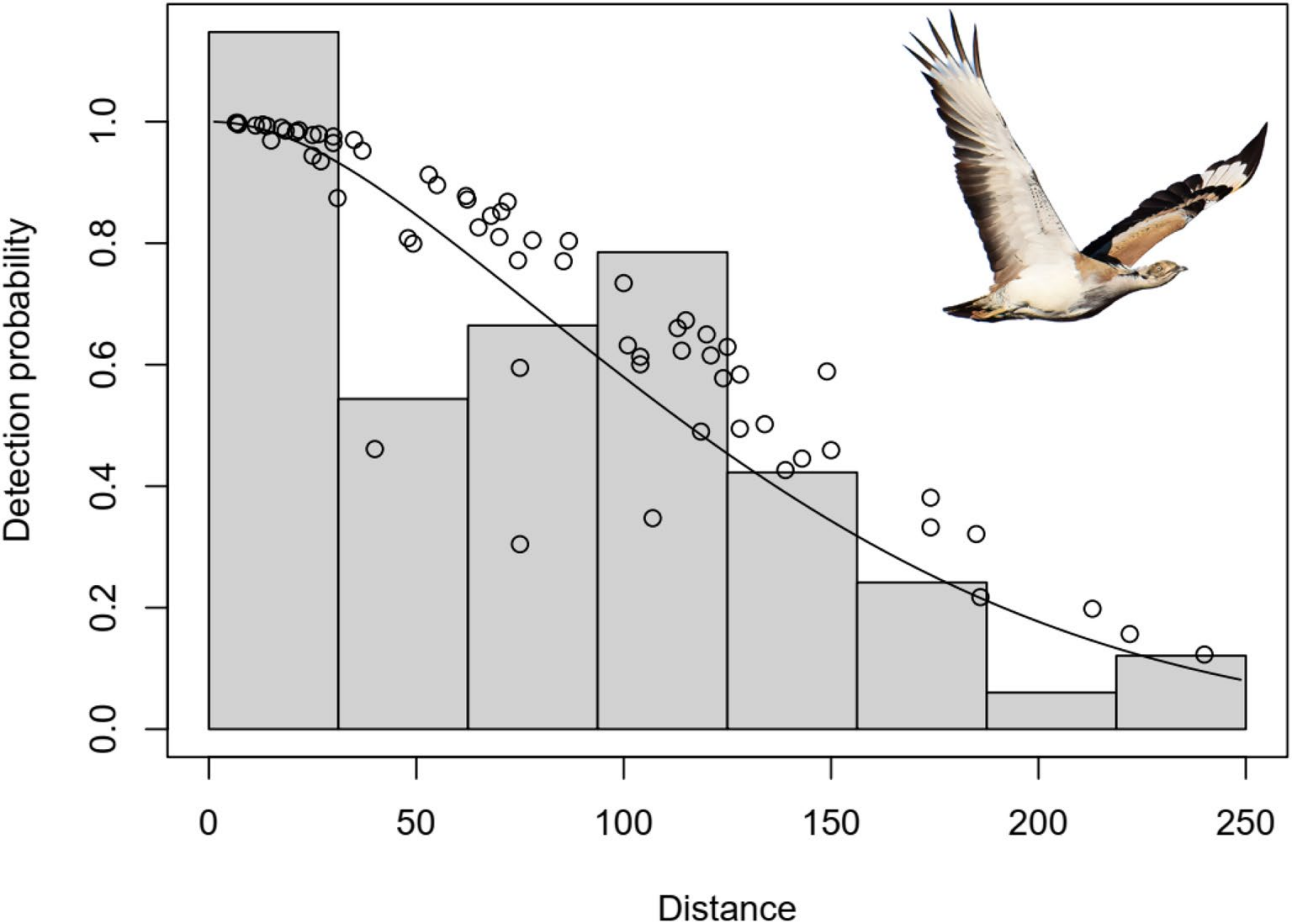
Detection function of the Multi‐Covariate Distance Sampling (MCDS) model incorporating fine gravel percentage and average vegetation height as covariates for the Asian houbara.

Between autumn 2022 and winter 2023, we conducted 223 line‐transect surveys across approximately 10,000 km^2^ of the study area (Figure 1), totaling 1449 km of survey effort (875 km in 2022; 574 km in 2023). During this period, 205 individuals were recorded in 67 distinct groups. The overall encounter rate was 0.12 groups per kilometer, with an average group size of 3.06 individuals. Among the candidate CDS models, the uniform key function with cosine adjustment terms provided the best fit, as supported by the Cramér–von Mises test (*p* = 0.92), although all models showed acceptable performance (Table 1). Detection probabilities estimated from this model were consistent with those obtained from the final MCDS model. The best‐supported MCDS model (FN + HT) incorporated fine gravel percentage (*β* = 0.005; SE = 0.005) and average vegetation height (*β* = −0.004; SE = 0.004) as covariates on detectability (Figure 2). While AIC‐based model selection favored the inclusion of these covariates and improved overall model fit, the effect sizes were small with standard errors approximately equal to the coefficients, indicating limited statistical support for strong individual effects. Nevertheless, detection probabilities from this model were consistent with those obtained from the final CDS model. Importantly, across all three modeling approaches used to assess habitat preferences (RF, GAM, and GLM), fine gravel percentage and vegetation height consistently emerged as the most influential predictors. Although these covariates did not meaningfully improve detectability estimates in the MCDS model, their consistent importance in the habitat models suggests they remain ecologically relevant for houbara habitat use and merit consideration in future studies. Based on this model, the estimated population across the study area was 5293 individuals (95% CI: 3778–7473; SE = 933; CV = 0.18), with a mean density of 0.53 individuals/km^2^ (95% CI: 0.37–0.75). Stratified density estimates revealed 0.66 individuals/km^2^ in high‐quality habitat and 0.35 individuals/km^2^ in low‐quality areas. For comparison, the top‐ranked CDS model without covariates produced a similar mean density estimate (0.52 individuals/km^2^; 95% CI: 0.37–0.73) to that obtained from the MCDS model. This close agreement indicates that including fine gravel and vegetation height as detectability covariates had little influence on overall abundance estimates, suggesting that MCDS did not substantially improve model accuracy in this case.

**TABLE 1:** Summary of various detection functions applied to distance sampling data for estimating Asian houbara density.

Models	Formula	AICc	ΔAIC	p‐a	%CV	SE	95% CI		Cvm *p*	D/km^2^
							DLCL	DUCL		
*MCDS*										
Half‐normal	~FN + HT	703.43	0.0	0.50	18	0.09	0.37	0.74	0.72	0.53
Half‐normal	~FN + HT + CR + MM + SHB + BSH + SD	710.88	7.45	0.49	18	0.1	0.38	0.78	0.56	0.54
*CDS*										
Half‐normal	No covariates	706.92	1.58	0.53	18	0.09	0.37	0.73	0.91	0.52
Uniform	No covariates	706.86	1.43	0.64	15	0.08	0.38	0.69	0.92	0.51
Hazard‐rate	No covariates	707.05	3.61	0.63	17	0.07	0.31	0.61	0.66	0.44

### 3.2 Habitat Use

**FIGURE 3:**
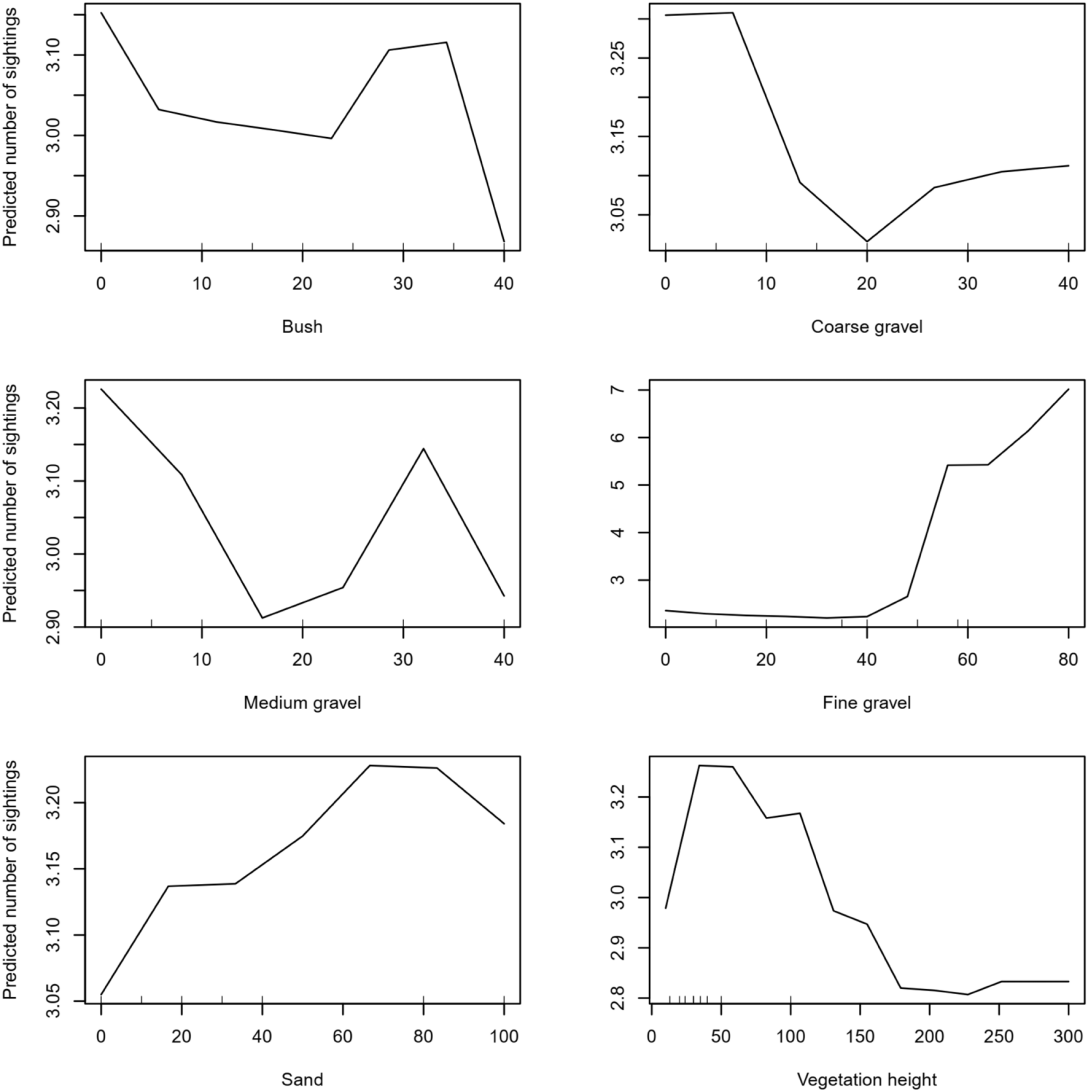
Response curves of the RF model for Asian houbara observations in relation to explanatory variables: Bush cover percentage, coarse gravel percentage, medium gravel, fine gravel percentage, average vegetation height, and sand cover.

Among the three modeling approaches used to assess habitat preferences, the RF model showed the highest predictive performance (*R* ^2^ = 0.86; ntree = 500; mtry = 6), outperforming the GAM (*R* ^2^ = 0.59) and the GLM (*R* ^2^ = 0.46). Despite differences in model performance, all three consistently identified fine gravel percentage and vegetation height as the most influential predictors. In the RF model, higher fine gravel cover was strongly associated with increased presence, while vegetation height showed a marked negative effect—particularly beyond ~2 m. Peak predicted densities occurred in areas with high fine gravel content and vegetation heights around 50 cm. Other environmental variables, including bush cover, sand content, and percentages of coarse and medium gravel, contributed minimally to model performance and had negligible influence on predicted occurrence (Table 2). Partial dependence plots from the RF model reinforced the importance of fine gravel (%IncMSE = 63.5) and vegetation height (%IncMSE = 15.6) as key predictors (Figure 3). Similar response trends were observed in the GAM and GLM models, underscoring the robustness of these relationships across analytical frameworks.

**TABLE 2:** Results of the Random Forest (RF) model for Asian houbara observations in relation to explanatory variables. The selection of these variables was based on ecological relevance and is supported by previous studies of houbara habitat selection (Carrascal et al. 2008; Hingrat et al. 2008; Koshkin et al. 2016; Ucero et al. 2025).

Variables	Importance	*R* ^2^	RMSE
*RF*			
Fine gravel	180.42		
Average vegetation height	44.31		
Medium gravel	25.24	0.86	0.84
Coarse gravel	14.12		
Bush cover	12.65		
Sand cover	7.11		

## 4 Discussion

As the global population of houbara continues to decline, accurate density estimates are essential for guiding conservation efforts (Kessler et al. 2024). While CDS remains widely used, it models detection solely as a function of distance. In contrast, MCDS incorporates habitat variables, enhancing model accuracy and ecological relevance (Crosbie et al. 2011; Zamboni et al. 2015). Despite its advantages, MCDS is still underutilized in avian research (Arandhara et al. 2020).

The largely open desert landscape, with only sparse *Haloxylon* patches, provided few obstacles to detecting birds directly on the transect line, supporting the assumption that detection probability at zero distance was effectively one. In addition, the shape of the detection function (Figure 2) provided no evidence of evasive movement prior to detection, indicating that these assumptions were reasonably met under our survey conditions. Precautions such as GPS logging of detections and noting flight directions when birds were flushed minimized the risk of double‐counting, making it unlikely that this source of bias affected abundance estimates. In our study, incorporating habitat covariates did not materially change abundance estimates compared to CDS models, indicating limited influence on detectability. Nonetheless, fine gravel percentage and vegetation height consistently emerged as important predictors in habitat models, highlighting their ecological relevance even if their effects on detectability were weak. The overall density estimate (0.53 individuals/km^2^) is lower than previous reports from the region (e.g., Hemami and Akbari‐Feizabadi 2007: 0.76; Aghanajafizadeh et al. 2012: 0.87), suggesting a continued decline. In contrast, lower spring population densities have been documented in parts of Uzbekistan (0.28 individuals/km^2^; Koshkin et al. 2016), highlighting regional variation linked to habitat quality. Although the confidence intervals around the population estimate (5293 individuals; 95% CI: 3778–7473) are relatively wide, this level of uncertainty is typical for large‐scale distance sampling surveys. At present, there is no standardized national protocol for estimating Asian houbara abundance in Iran, and available information is largely based on direct observations reported by Provincial Departments of Environment. These records, which represent observed numbers rather than population estimates, have documented between 2000 and 3400 houbara individuals nationwide over the past 4 years. In our line‐transect surveys, we directly observed 205 individuals within a 10,000 km^2^ area, whereas distance‐sampling analysis indicated a substantially larger population within the same area. This contrast illustrates that observed counts represent only a fraction of the true population size and suggests that national‐level population estimates are likely several‐fold higher than numbers currently recorded through direct observations. Although extrapolation to the national scale is inappropriate due to spatial variation in habitat suitability and distribution, these findings indicate that Iran may support a much larger houbara population than currently recognized, underscoring the country's potentially important role in the species' range‐wide conservation and the need for standardized, large‐scale monitoring.

Density estimates from the MCDS and CDS models were nearly identical, suggesting that weak effects of detectability covariates did not substantially influence final abundance estimates. This consistency reinforces confidence in the robustness of our results, though the relatively wide uncertainty intervals emphasize the need for continued monitoring, larger sample sizes, and potential future sensitivity analyses. Future surveys with larger sample sizes and repeated temporal coverage will be essential to reduce uncertainty and better inform conservation planning.

Habitat stratification within our study area revealed that densities were lower (0.35 individuals/km^2^) in zones dominated by dense vegetation or sandy terrain, and higher (0.66 individuals/km^2^) in areas characterized by moderate vegetation and gravel substrates. Detection probability also declined with increasing distance from the transect line, with most detections occurring within 150 m—likely a result of visual obstruction from vegetation or terrain complexity (Buckland et al. 2015). Our final detection model, which incorporated key habitat features, further validated the utility of habitat‐based covariates in improving model fit.

Habitat structure emerged as a central driver of houbara distribution. Individuals tended to occupy areas with intermediate vegetation height; likely striking a balance between concealment and visibility for predator avoidance. These findings align with previous studies reporting higher densities in bush‐steppe habitats and lower densities in shrub‐steppe or sandy environments (Aghainajafi‐Zadeh et al. 2010; Koshkin et al. 2016). Vegetation height may be especially important for nesting females, with reported nest‐site heights (~47.2 cm) corresponding closely to the head height of incubating birds—allowing visual awareness while remaining concealed (Guilherme et al. 2018; Ucero et al. 2025). Similar patterns have been documented in other ground‐nesting birds such as the little bustard ( *Tetrax tetrax* ), Great Bustard ( *Otis tarda* ), Crested Lark ( *Galerida cristata* ), and Corn Bunting ( *Miliaria calandra* ) (Rodrigues 1994; Morales et al. 2008, 2013; Magana et al. 2010; Silva et al. 2010; Ponce et al. 2018). While some studies have emphasized vegetation height in houbara habitat selection (Carrascal et al. 2008; Koshkin et al. 2016), others have overlooked it (e.g., Seddon and Van Heezik 1999; Hingrat et al. 2008). Our results reinforce its ecological significance and highlight its value in predictive modeling.

Among the models tested, RF provided the most accurate predictions, capturing complex, nonlinear interactions (Liaw and Wiener 2002). GAM also performed well, underscoring its relevance for ecological data with nonlinear responses (Guisan and Zimmermann 2000; Aarts et al. 2008). Across models, fine gravel emerged as a consistently strong predictor of occurrence, supporting earlier findings (Koshkin et al. 2016). Areas with moderate vegetation (~50 cm) and high fine gravel cover had the highest predicted occurrence, likely due to improved camouflage and mobility.

Despite logistical challenges, establishing a coordinated, long‐term monitoring program should be a central priority for Asian houbara conservation in Iran, particularly given the country's large size and environmental heterogeneity. This study provides the first large‐scale, conservation‐oriented assessment of houbara populations in Isfahan Province and demonstrates the feasibility of applying standardized line‐transect distance sampling to generate robust baseline data for repeated monitoring. Our findings translate directly into management guidance by identifying habitat features associated with higher houbara occurrence, highlighting the importance of conserving open wintering habitats characterized by fine gravel substrates and moderate vegetation height. Implementing standardized survey protocols across key regions would enable reliable detection of population trends, facilitate evaluation of management effectiveness, and strengthen national population assessments. Future conservation efforts should integrate long‐term monitoring with targeted habitat management, expansion of surveys to other regions of Iran, and spatially explicit analyses to support adaptive management and evidence‐based policy decisions.

## Author Contributions

**Reyhaneh Miranzadeh‐Mahabadi:** data curation (equal), formal analysis (lead), visualization (lead), writing – original draft (lead), writing – review and editing (equal). **Mahmoud‐Reza Hemami:** conceptualization (equal), formal analysis (supporting), methodology (equal), supervision (lead), writing – original draft (supporting), writing – review and editing (equal). **Hossein Bashari:** validation (equal), writing – review and editing (equal). **Mostafa Abyareh:** data curation (equal), writing – review and editing (equal). **Mohsen Ahmadi:** conceptualization (equal), formal analysis (supporting), methodology (equal), writing – review and editing (equal).

## Conflicts of Interest

The authors declare no conflicts of interest.

## Supporting information


**Data S1:** Supporting Information.

## Data Availability

The data supporting the findings of this study are publicly available in Dryad at https://doi.org/10.5061/dryad.hmgqnk9z4.
